# Protecting the endothelial glycocalyx in COVID-19

**DOI:** 10.1371/journal.ppat.1012203

**Published:** 2024-05-16

**Authors:** Emira Adam Tay, Vimmal Vijayakumar, Raika Francesca Morales, Ee Soo Lee, Andrew Teo

**Affiliations:** 1 School of Applied Science, Republic Polytechnic, Singapore, Singapore; 2 National Centre for Infectious Diseases, Singapore, Singapore; 3 Lee Kong Chian School of Medicine, Nanyang Technological University, Singapore, Singapore; 4 School of Pharmacy, University of Nottingham Malaysia, Selangor, Malaysia; 5 Department of Medicine, The Doherty Institute, University of Melbourne, Melbourne, Australia; University of Iowa, UNITED STATES

## Introduction

COVID-19 is a respiratory infection caused by the highly transmissible SARS-CoV-2. While most infected patients exhibit mild to moderate symptoms, a minority may develop severe vascular damage, which can be fatal. One example of this damage is disruption to the endothelial glycocalyx (EG) layer. EG alterations have been implicated in microcirculation changes and severe endothelial dysfunction in COVID-19 [1,2]. Current evidence suggests that host–pathogen interactions, such as hyperimmune activation or persistent presence of viral antigens, are associated with EG damage and may potentially bring about continuous endothelial dysfunction even after clinical recovery. Notably, the glycocalyx is also present on the epithelium, but we will focus on the EG and its implication on vascular health in COVID-19. Additionally, we will propose possible mediators that are linked with EG damage and suggest therapeutic agents that could aid EG restoration and protection.

## Damage to the endothelial glycocalyx layer may affect vascular health

**Fig 1:**
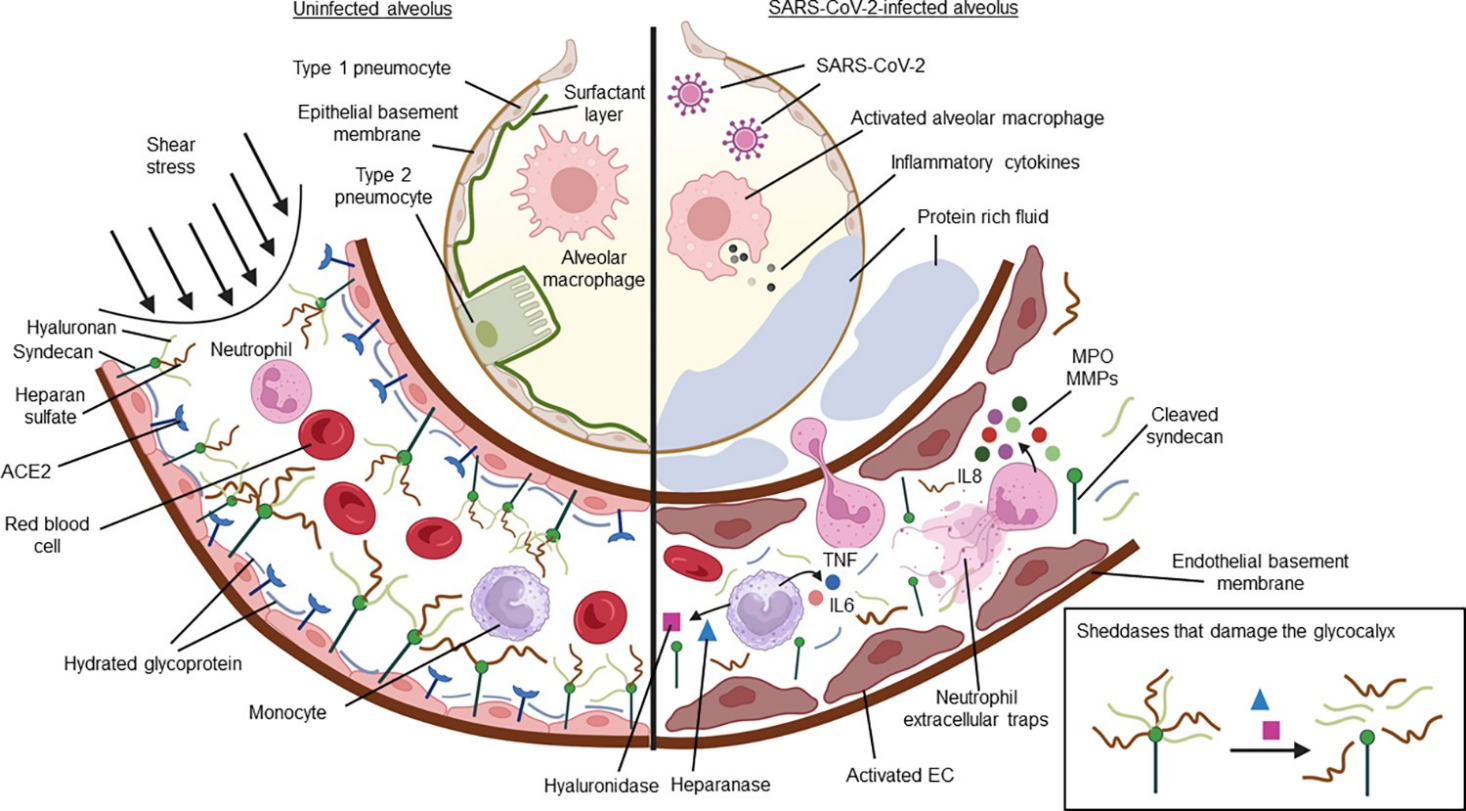
Diagram depicting the lower respiratory tract, uninfected alveolus (left panel) and infected alveolus (right panel). **Uninfected condition (left panel)**, in an uninfected condition, the EG regulates the mechanotransduction of flow-induced shear stress in the vascular lumen, promoting a homeostatic environment. SARS-CoV-2 infection (right panel), SARS-CoV-2 infection activates the immune defence to eliminate the pathogen. In the alveolus, activated alveolar macrophages release inflammatory cytokines, driving a local inflammatory response. If unresolved, this inflammation may damage the alveolus. Damaged to the (EG) lining promotes neutrophil migration to the site of infection. Activated neutrophils release antimicrobials such as myeloperoxidase (MPO) and neutrophil extracellular traps, which can damage the glycocalyx. Other sheddases including matrix metalloproteinase (MMP), heparanase, and hyaluronidase, are also released and may further disrupt the glycocalyx integrity. These events damage the pulmonary vasculature, which can lead to fluid buildup in the lungs (pulmonary edema) and respiratory failure. Importantly, a damaged EG also affects mechanotransduction in the circulatory system, potentially altering endothelial cell functions in various organs. This may give rise to multiorgan failure, which is fatal in severe COVID-19 cases. Created with Biorender.

The EG layer, composed of a network of membrane-bound filament-like glycoproteins lining the luminal surface of the endothelium, functions to maintain vascular haemostasis and modulate leukocyte–endothelial interactions, blood flow, and vascular permeability (reviewed in [3]). These glycoproteins include heparan sulfate (HS), syndecan and hyaluronan, which are susceptible to inflammatory insults. Disruption to these glycoproteins is hypothesised to precede endothelial injury and weakens the structural integrity of the vasculature. These disruptions may give rise to increased pulmonary permeability, disrupt shear stress sensing of the endothelial cells, and affect physiological flow (Fig 1). In support, observational studies have shown that a thinner vascular EG, quantified by video imaging, correlates with significant reductions in vascular density and reduced red blood cell velocity, which is associated with increased disease severity [1,2]. Additionally, severe COVID-19 cases exhibit increased circulation of EG proteoglycans (syndecan-1, HS, and hyaluronan) and markers of endothelial injury (vascular endothelial growth factors, angiopoietin-1, and soluble thrombomodulin), compared to non-severe and uninfected cases [1,2,4,5]. Together, these findings suggest that in severe COVID-19, endothelial injury is closely linked to the integrity of the EG, and damage to the endothelial lining may lead to severe vascular complications (reviewed in [6]). Interestingly, endothelial dysfunction may persist in some recovered individuals, as shown by increased levels of circulating endothelial cells and syndecan-1 shedding [5,7]. This has been attributed to unresolved viral presence and/or inflammation, contributing to prolonged EG damage and dislodgement of endothelial cells from injured blood vessels [7–9]. The exact cause of EG damage or prolonged damage in COVID-19 remains to be determined, but current evidence suggests that host–pathogen interactions may play a role in EG injury.

## Proteoglycans of endothelial glycocalyx may promote spike protein interaction with endothelial cells

The spike protein on the virion is known to bind to the angiotensin converting enzyme 2 (ACE2) receptor to initiate cellular entry, and HS has been proposed to mediate initial virus-ACE2 interaction [10]. It is worth noting that the majority of cellular surfaces are covered by proteoglycans, making them likely targets for viruses like coronaviruses to exploit as attachment factors and receptors for entry [11]. For example, in laboratory settings, the interaction between spike protein and HS led to conformational changes in the virion that promoted binding to ACE2. Furthermore, SARS-CoV-2 binding is greatly reduced upon cleavage of HS [10]. This demonstrates a complex interplay, where cleaving of HS is likely to reduce the availability of HS that promotes early attachment but could also increase ACE2 expression, potentially making cells more susceptibility to direct spike protein interaction [12]. Whether increased ACE2 exposure translates to higher SARS-CoV-2 infectivity in the epithelium is unclear, but peak viral load has been correlated with disease severity [13]. Further investigations into whether synthetic HS or ACE 2 can block viral attachment, thus, reducing infection are warranted as a potential prophylaxis against coronavirus infection [14,15].

## Activated host responses contribute to the damage of the endothelial glycocalyx

The specific mechanism responsible for EG degradation is not fully understood. It is postulated that elevated levels of sheddases, such as heparanase, hyaluronidase, and matrix metalloproteases (MMP) can promote EG shedding and endothelial cell activation (Fig 1) [4,16–18]. Notably, heparanase and hyaluronidase are known mammalian enzymes that cleave HS and HA, respectively (reviewed in [19]). Damage to the EG exposes leukocyte-binding receptors (ICAM-1, VCAM-1), which are usually embedded within the EG. This can increase leukocyte extravasation, triggering a cascade of downstream activities that likely further damage the EG and endothelial cells [20]. To corroborate, in autopsied COVID-19 lung tissues, vascular and endothelial pathologies coupled with leukocyte infiltration have been observed [21]. This further supports the notion that uncontrolled leukocyte extravasation, along with EG and endothelial cell damage, can cause vascular injury. While in other viruses such as dengue virus, secreted viral antigen has been demonstrated to damage the EG (reviewed in [22]). However, current evidence does not support this hypothesis in SARS-CoV-2.

Activated leukocytes, such as neutrophils, release antimicrobial agents like myeloperoxidase (MPO), which has been shown to correlate with syndecan-1 shedding in COVID-19 [5]. MPO has also been proposed to bind to HS, resulting in the weakening of EG structure and subsequent damage [23]. Similarly, increased inflammatory mediators including IL-6, IL-8, and TNF-ɑ have been associated with elevated plasma concentrations of syndecan-1, hyaluronan, and HS in severe COVID-19 [4,16]. These cytokines may also act as stimuli to affect the integrity of the EG. For example, IL-6 binds to glycosaminoglycans, which can either weaken the EG structure and/or promote EG degradation via paracrine effects [24]. On the other hand, glycosaminoglycan fragments such as hyaluronan, isolated from COVID-19 subjects, have been demonstrated to bind to hyaluronan receptors on endothelial cells. This results in the activation of Rho-associated protein kinase signalling, a known pathway that triggers EG disruption [4]. Together, various factors can either directly or indirectly affect the integrity of EG. Therefore, minimising EG damage presents a potential pathway to reducing the risk of severe pathology.

## Protecting the endothelial glycocalyx may reduce disease severity

A damaged EG contributes to impaired vascular health, and several classes of interventions have the potential to minimise COVID-19-associated EG damage (Table 1). In vitro studies have shown that neutralising antibodies against the spike protein block binding of SARS-CoV-2 on HS and ACE2, thereby reducing infection and inflammation [25,26]. Additionally, low molecular weight heparin (LMWH) was shown to bind to the spike protein, which led to conformational change, that interfered with virus attachment to HS and reduced viral entry [25,27]. Moreover, LMWH was associated with a significant reduction in IL-6 levels in COVID-19 patients and appeared to have inhibitory properties against heparanase [16,28]. Similarly, tocilizumab, an IL-6 receptor blocker has been demonstrated to improve COVID-19 outcomes [29]. Treatment with tocilizumab protected against EG degradation in vivo, suggesting that reducing IL-6 function may reduce downstream inflammatory pathways that are associated with EG degradation [30]. Dexamethasone, a corticosteroid that has anti-inflammatory properties, is used in COVID-19 treatment. In septic mouse models, dexamethasone significantly reduced MMP activities and protected against EG damage [31]. In a COVID-19 clinical study, dexamethasone treatment was associated with significant reductions in C-reactive protein and markers of endothelial injury. However, whether the improved outcomes were due to MMP reduction that protected against EG degradation in COVID-19 remains uncertain [32]. Overall, reducing infection and inflammation is likely to protect against EG degradation [4,5,17,25].

**Table 1:** Potential compounds that may either reduce and/or restore the endothelial glycocalyx.

Therapies that may potentially protect against endothelial glycocalyx damage via the attenuation of inflammation		
Therapy	Purpose	Effects
Approved COVID-19 vaccines	To promote the development of neutralising antibodies against SARS-Cov-2	To neutralise the virus, thus reducing infection and inflammation [45].
Tocilizumab	Humanised monoclonal antibody that targets against interleukin-6 receptor	Inhibits IL-6 binding to its receptor and reduces inflammatory activation [30].
Baricitinib	A Janus kinase inhibitor that reduces cytokine signalling cascade	Suggested to reduce inflammatory mediators including neutrophil granules (MPO, MMP), TNF-α, and IL-6 [46].
Dexamethasone	A corticosteroid that has anti-inflammatory property	Proposed to reduce inflammation including reduction of serum C-reactive protein and possibly matrix metalloproteases [31,32].
Low molecular weight heparin (LMWH)	Proposed to bind to spike protein on the virus and demonstrated anti-inflammatory effects	Demonstrated to interfere with virus binding on host receptors and to reduce inflammation [17,25,27,28].
Myeloperoxidase (MPO) inhibitors	To inactivate MPO activity	Myeloperoxidase has been proposed to weaken the endothelial glycocalyx integrity. Inhibiting its activity may reduce glycocalyx disruption [5,23].
Rho kinase (ROCK) inhibitor	To inhibit Rho kinase activity	Activated ROCK triggers glycocalyx disruption. ROCK inhibition may reduce disruption [4].
**Therapeutics that may aid the restoration of the endothelial glycocalyx**		
**Therapy**	**Purpose**	**Effect**
Imatinib	Tyrosine kinase inhibitor	Demonstrated to improve endothelial glycocalyx thickness and endothelial functions. Mechanisms remain unclear [33,34].
Fucoidan	Heparan sulfate mimetic that promotes heparan sulfate and hyaluronan synthesis	Proposed to reduce endothelial activation and restore endothelial glycocalyx thickness [37,39].
Rhamnan Sulphate	Heparan sulfate mimetics that can reduce inflammation	Proposed to reduce leukocyte adhesions and promote endothelial glycocalyx regeneration [41].
Sulodexide	Purified heparin and dermatan sulfate	Demonstrated to restore endothelial glycocalyx in septic mice and reduce hyaluronidase activity [42,43].
Fresh frozen plasma and albumin	Plasma proteins that are involved in repairing the endothelial glycocalyx	Commonly used in blood volume resuscitation and has been demonstrated to improve endothelial glycocalyx thickness in septic conditions [44].
Fingolimod (FTY720)	Sphingosine 1 phosphate (S1P) receptor agonist	S1P is a sphingolipid that binds to S1P receptors that may improve endothelial glycocalyx integrity. Proposed mechanisms include inhibiting syndecan-1 shedding and reducing matrix metalloproteinase activity [47]. Its effect on COVID-19 is currently unclear [48].

## Possible therapies that restore the endothelial glycocalyx

Complementary therapies that minimise inflammatory-induced EG degradation are therapeutics that aid the regeneration of EG (Table 1). In COVID-19, treatment with a tyrosine kinase inhibitor, imatinib, has been demonstrated to improve endothelial barrier functions [33]. In an in vitro model, treating compromised endothelial cells with imatinib was shown to improve EG thickness and restore EG functions, although the precise mechanism remains to be determined [34]. Extracts from marine algae, fucoidan and rhamnan sulfate (RS), which are safe in humans [35,36], have been shown to restore EG both in vivo and in vitro. Notably, fucoidan, an HS mimetic, reduced endothelial activation and promoted EG restoration on endothelial cells previously treated with COVID-19 serum [37]. Fucoidan is proposed to contain amino acids capable of stimulating HS and hyaluronan synthesis (reviewed in [38]). Additionally, fucoidan improved EG thickness in aged mice (where thinning of EG is common due to aging) and increased nitric oxide (NO) bioavailability [39]. NO is a potent vasodilator that promotes blood flow and reduced NO production increases the risk of endothelial dysfunction (reviewed in [40]). Similarly, RS, another HS mimetic, limited leukocyte adhesion, increased HS coverage on endothelial cells and promoted EG regeneration in a study on vascular inflammation [41]. Sulodexide, a mixture of heparin and dermatan sulfate, is used to treat peripheral vascular disease. Administrating sulodexide to septic mice restored EG and reduced vascular permeability [42]. Sulodexide was also reported to reduce hyaluronidase activity in type 2 diabetic subjects, which was associated with increased EG regeneration [43]. Besides these repurposed compounds, fluid therapy with fresh frozen plasma (FFP) and albumin are often used in blood volume resuscitation in various vascular conditions. For instance, FFP contains most of the plasma proteins needed for EG maintenance, whereas albumin is a major component in plasma and both interventions have been shown to improve EG thickness in septic conditions [44]. Additionally, albumin carries sphingosine-1-phosphate, a bioactive lipid metabolite that has been demonstrated to stabilise and prevent EG shedding (reviewed in [44]). Despite these promising therapies to aid the restoration of the EG, a gold standard treatment has yet to be translated clinically.

## Conclusions

Available evidence suggests that EG disruption contributes to severe COVID-19 outcomes. The therapeutic candidates presented here offer promising avenues for developing interventions that can protect and restore the EG. Further studies are urgently needed to evaluate the efficacy and safety of these candidates, whether alone or in combination, to prepare for future outbreaks and improve patient outcomes for future outbreaks.
